# Integrating past experiences

**DOI:** 10.7554/eLife.106291

**Published:** 2025-03-27

**Authors:** Thomas MW Leir, Matthew PH Gardner

**Affiliations:** 1 Department of Psychology, Faculty of Arts and Sciences, Concordia University Montreal Canada

**Keywords:** fear, memory, mediated learning, chaining, integration, sensory stimuli, Rat

## Abstract

New results help address a longstanding debate regarding which learning strategies allow animals to anticipate negative events based on past associations between sensory stimuli.

**Related research article** Wong FS, Thomas AB, Killcross S, Laurent V, Westbrook RF, Holmes NM. 2025. Integration of sensory and fear memories in the rat medial temporal lobe. *eLife* **13**:RP101965. doi: 10.7554/eLife.101965.

The brain’s ability to integrate distinct memories is critical for forming accurate predictions in unfamiliar situations. This process can be studied in the laboratory using a three-stage preconditioning task (Brogden, 1939). First, animals learn to associate two ‘neutral’ stimuli through a sequential presentation, such as a sound followed by a light. Next, the latter stimulus (for instance, the light) is followed by a motivationally meaningful experience, such as a fear-inducing foot shock. The final stage tests how the animals respond to the neutral stimulus not directly associated with the negative experience (in this case, the sound). Typically, the animals behave as if this signal also predicts the foot shock, showing that they have integrated information from the neutral association (between sound and light) with that of the motivational association (between light and shock).

Two conflicting perspectives exist on how this integration occurs (Gostolupce et al., 2022; Holmes et al., 2022; Rizley and Rescorla, 1972). The mediated theory proposes that integration occurs at the second stage: the neutral association formed in the first stage allows the animals to ‘picture’ the sound signal as the light is shown during the second stage, permitting development of an association between the sound and the shock (Iordanova et al., 2011; Wong et al., 2019). Alternatively, integration could occur during the test phase through a process known as chaining; the animals ‘think’ of the light when they hear the sound signal and therefore recall the shock, chaining together these separate sound-light and light-shock associations. Now, in eLife, Nathan Holmes and colleagues from the University of New South Wales – including Francesca Wong as first author – report that both chaining and mediated learning are plausible mechanisms for integrating this information, but that each mechanism is used in different situations (Wong et al., 2025).

The team focused on two brain regions: the perirhinal cortex, which forms the neutral stimulus associations, and the basolateral amygdala, which supports the fear associations (Holmes et al., 2013). In 2019, three members of the team showed that neural plasticity in the perirhinal cortex depends on a receptor known as NDMA and is required during the second stage of preconditioning. This indicated that integration could potentially take place through mediated learning during this stage (Wong et al., 2019).

Building on this previous work, Wong et al. used the sensory preconditioning task to train rats either extensively or briefly during stage one, which meant that the two groups differed in the number of sound-light pairings they experienced. NMDA receptors in the perirhinal cortex were then pharmacologically blocked during stage two when the light was paired with the foot shock, which should interfere with mediated conditioning, but not the chaining method. Supporting their previous findings, this resulted in a decreased response to the sound stimulus during the test phase. However, this only occurred in rats for which stage one had been brief. This suggests that rats with limited sound-light training used mediated conditioning, while those with extensive training likely engaged in the chaining method at test (Figure 1).

**Figure 1.**
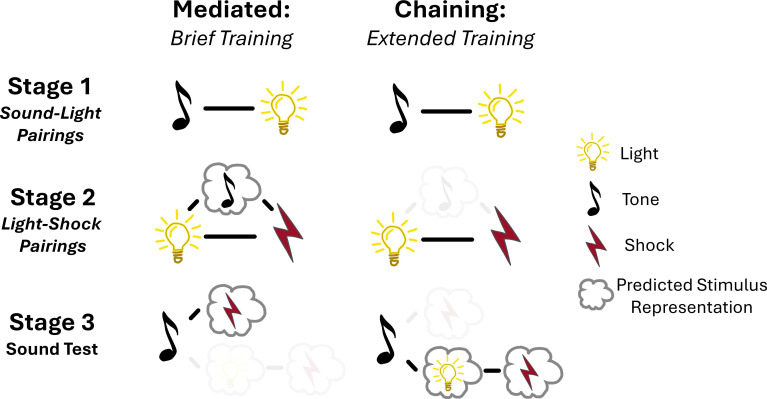
Proposed mechanisms of learning during brief or extended preconditioning training. Wong et al. used a preconditioning task to study how rats learn. In stage 1 (top), rats were exposed to a neutral stimulus pairing of sound followed by a light. In stage 2 (middle), the light was followed with an electric shock, which represents a negative outcome. In stage 3 (bottom), rats were presented with the neutral sound signal from stage 1. The rats associated this neutral stimulus with the electric shock and acted fearfully, even though the sound stimulus had not previously been presented with the electric shock. The stimuli were counterbalanced across all experiments, so that half of the rats in each group received the training in the order shown in this figure and half were trained with light-sound pairings in stage 1, sound-shock pairings in stage 2 and a light test in stage 3. Varying how many times the rats are presented with the neutral stimulus pairings in Stage 1 dictates the method they use to integrate associations between the neutral stimuli and the subsequent unpleasant electric shock. After brief training (left) rats use a mediated learning mechanism during stage 2 to associate the sound with the electric shock. With extended training (right), rather than carrying out mediated learning during stage 2, associations are made during the test phase (stage 3), using the chaining technique. This supports the theory that the sound elicits a prediction of the light and subsequent shock through reactivation of the memories stored during stage 1 and stage 2, respectively.

Next, to investigate whether communication between the perirhinal cortex and the basal amygdala was required for this integration, Wong et al. inactivated each region on opposite sides of the brain. This standard procedure for determining functional connectivity between brain regions relies on the observation that connections are primarily within a single hemisphere. If communication between the brain regions is required, inactivating the regions in different hemispheres prevents information from being processed in either hemisphere. The experiments revealed that communication was always required for integration. When this process took place, however, varied in line with the findings from the previous experiments. If stage one training was short, then communication between the two regions was required during stage two, when mediated learning theoretically occurs. On the other hand, if stage one was extended, this communication was required during the test stage, when chaining theoretically occurs.

Taken together, these findings help to reconcile the debate between mediated integration and chaining. However, it remains unclear why the training duration affects the mechanism of learning. Wong et al. suggest that the additional training in stage one strengthens the temporal relationship between the neutral stimuli (sound followed by light). This prevents mediated learning, which requires a reverse temporal sequence (where exposure to light triggers the thought of sound) to facilitate the sound-shock association (Figure 1). On the other hand, this additional training makes chaining possible, since temporal associations can strongly predict the order of events. Future research is required to determine whether this argument can explain the effect or whether other mechanisms may contribute to this type of learning.
